# Chromosome-level reference genomes of two imperiled desert fishes: spikedace (*Meda fulgida*) and loach minnow (*Tiaroga cobitis*)

**DOI:** 10.1093/g3journal/jkad157

**Published:** 2023-07-19

**Authors:** Nicolas M Alexandre, Alexander C Cameron, David Tian, Kamalakar Chatla, Sree R R Kolora, Noah K Whiteman, Thomas F Turner, Peter N Reinthal

**Affiliations:** Department of Integrative Biology, University of California, Berkeley, CA 94720, USA; Museum of Vertebrate Zoology, Berkeley, CA 94720, USA; Museum of Southwestern Biology and Department of Biology, University of New Mexico, Albuquerque, NM 87131, USA; Department of Integrative Biology, University of California, Berkeley, CA 94720, USA; Museum of Vertebrate Zoology, Berkeley, CA 94720, USA; Department of Integrative Biology, University of California, Berkeley, CA 94720, USA; Museum of Vertebrate Zoology, Berkeley, CA 94720, USA; Department of Integrative Biology, University of California, Berkeley, CA 94720, USA; Museum of Vertebrate Zoology, Berkeley, CA 94720, USA; Department of Integrative Biology, University of California, Berkeley, CA 94720, USA; Museum of Vertebrate Zoology, Berkeley, CA 94720, USA; Museum of Southwestern Biology and Department of Biology, University of New Mexico, Albuquerque, NM 87131, USA; Department of Ecology and Evolutionary Biology, University of Arizona, Tucson, AZ 85721, USA

**Keywords:** conservation genomics, Cypriniformes, freshwater fish, Leuciscidae, minnows, wildlife management

## Abstract

North American minnows (Cypriniformes: Leuciscidae) comprise a diverse taxonomic group, but many members, particularly those inhabiting deserts, face elevated extinction risks. Despite conservation concerns, leuciscids remain under sampled for reference assemblies relative to other groups of freshwater fishes. Here, we present 2 chromosome-scale reference genome assemblies spikedace (*Meda fulgida*) and loach minnow (*Tiaroga cobitis*) using PacBio, Illumina and Omni-C technologies. The complete assembly for spikedace was 882.1 Mb in total length comprised of 83 scaffolds with N50 = 34.8 Mb, L50 = 11, N75 = 32.3 Mb, and L75 = 18. The complete assembly for loach minnow was 1.3 Gb in total length comprised of 550 scaffolds with N50 = 48.6 Mb, L50 = 13, N75 = 42.3 Mb, and L75 = 20. Completeness assessed via Benchmarking Universal Single-Copy Orthologues (BUSCO) metrics using the Actinopterygii BUSCO database showed ∼97% for spikedace and ∼98% for loach minnow of complete BUSCO proportions. Annotation revealed approximately 32.58 and 29.04% of spikedace and loach minnow total genome lengths to be comprised of protein-coding genes, respectively. Comparative genomic analyses of these endangered and co-distributed fishes revealed widespread structural variants, gene family expansions, and evidence of positive selection in both genomes.

## Introduction

Leuciscidae (Cypriniformes: Cyprinoidei) is a species-rich family of cypriniform fishes that are a major component of North American freshwater fish communities (Schönhuth *et al*. 2018). Ecological diversity of leuciscids is prominent, and uncovering the evolutionary processes that shaped this diversity is an area of rich research (Gidmark and Simons 2014; Ross 2019). However, studies investigating genomic evolution among leuciscid fishes have lagged and remain limited (Gold and Amemiya 1987; Gold *et al*. 1990). Leuciscids from the western United States and Mexico are of particular interest given a unique interplay of biogeography and paleo-climatic changes that produced high rates of endemism and monotypic lineages (Smith 1981; Mayden 1988; Smith *et al*. 2002). Further, fishes of the desert southwest are among the most endangered groups of vertebrates (Jelks *et al*. 2008; Burkhead 2012), and the development of genomic resources is a major step toward advancing species management and conservation (Turner *et al*. 2021; Osborne *et al*. 2022). The proliferation of high-throughput and long-read sequencing technologies has the potential to fill key knowledge gaps regarding genomic evolution through comparative genomics and could substantively improve conservation prospects for imperiled taxa within this group.

Spikedace (*Meda fulgida*) and loach minnow (*Tiaroga cobitis*) are monotypic genera within Leuciscidae endemic to the Gila River Basin (Arizona and New Mexico), a major tributary of the Lower Colorado River system. Both species have overlapping but restricted geographic distributions as they have been extirpated from more than 80% of their historic range (Propst 1999). Consequently, each is recognized as endangered under the Endangered Species Act (*Endangered and threatened wild- life and plants; endangered status and designations of critical habitat for spikedace and loach minnow: final rule* 2012). Despite their conservation status and rarity, the general biology and ecology of both species are well detailed. Loach minnow is a benthic, obligate riffle species, reflected in its morphological appearance (e.g. large pectoral fins and reduced swim bladder) and relies on interstices of stream substrate within riffle microhabitat for foraging and reproduction (Propst *et al*. 1986). Spikedace presents a streamlined morphology and occupies a wider variety of microhabitats compared with loach minnow (Stefferud *et al*. 2011), but still exhibits affinity for habitats with higher water velocity (Barber *et al*. 1970; Propst *et al*. 1986; Hedden *et al*. 2022). Spikedace and loach minnow share general life history aspects, in that both are small-bodied (max standard length = 76 mm; >70 mm (Hendrickson 2011)), short-lived (24 months (Propst *et al*. 1986); 36 months (Propst and Bestgen 1991)), and are sexually dimorphic (i.e. male breeding coloration (Barber *et al*. 1970; Propst and Bestgen 1991)). However, it is not known how evolution has shaped the genomes of these relict species relative to other cypriniforms.

In this paper, we report chromosome-scale reference genome assemblies for spikedace and loach minnow using a combination Pacific Biosciences (PacBio) and Illumina and Hi-C (Omni-C) sequencing technologies. We also provide transcriptome-guided annotation of both genome assemblies. Using these novel reference assemblies, we investigated genome synteny, gene family expansions, and signatures of selection in spikedace and loach minnow.

## Material and methods

### Biological materials

Single individuals of spikedace and loach minnow of unknown age or sex were collected from upper Aravaipa Creek, (12S 556711 3638121), in Graham County, Arizona. Both species were netted live and euthanized using MS222. After internal organs were removed, each specimen was placed in a freezer-safe 2-mL screw cap cryovial and flash-frozen in the field in liquid nitrogen and stored in dry ice. A tissue subsample was sent to Dovetail Genomics to generate Omni-C libraries.

Two loach minnow individuals (MSB 109795-96), 1 male and 1 female, were collected for RNA sequencing for downstream genome annotation. Gut contents were removed from both individuals, then, the head and ovaries or testes were dissected and separated into 2-mL cryovials, flash-frozen, then filled with RNALater and stored at −80°C.

Three spikedace individuals (MSB 107682-83) were collected, 2 males and 1 female, and had their jaw and gastro-intestinal tract removed to prevent contamination. All individuals were flash-frozen in liquid nitrogen and stored in RNALater on dry ice.

Each loach minnow individual was separated into head and body for separate sequencing libraries including the brain and sex organs, respectively. Only head tissue was used for the 3 spikedace individuals. Tissues for all samples were then homogenized using a bead-beating step prior to RNA extraction with the RNeasy Plus Universal Midi Kit.

### Nucleic acid library preparation and sequencing

Genomic library preparation and genome sequencing were performed on the PacBio II Sequel instrument. Genome sizes were expected to range between 1 and 1.5GB based on *C*-values previously obtained for cyprinid fishes at genomesize.com (“Animal genome size database:: Home”). We extracted genomic DNA for genome sequencing using tissue from both individuals using a phenol extraction and 2 Salt:PCI cleanups. We then prepared a single library for each species using the SMRTbell Express Template Preparation Kit 2.0. We used a subset of the DNA extract from spikedace to generate paired-end end read data for error-correcting. A single library was generated for this purpose using the Roche Kapa Hyper Prep Kit.

RNA was isolated for both loach minnow and spikedace with the RNeasy Plus Universal Midi Kit, using ceramic mortar and pestle for disruption of tissue. Extracts from head and ovary/testes were then pooled for each individual. Libraries were generated using the Roche Kapa Hyper Prep kit.

Sequence data were generated from a single cell for spikedace and 2 cells for loach minnow on the PacBio Sequel II. Polymerase reads for Spikedace PacBio library averaged 19 kb while polymerase N50 was at 35.2 kb with N50 of subreads at 31.3 kb. We generated 65 Gb of data in a single cell of the PacBio Sequel II. Polymerase reads for the loach minnow library were 84 kb for the first cell and 95 kb for the second cell. For each cell, polymerase N50 was at 175 and 184 kb, with N50 of subreads at 14 and 16 kb, respectively. We generated 161 Gb of data across on 2 cells of the PacBio Sequel II. An additional 10 Gb of paired-end read data were generated on the NovaSeq 6000 S4 for spikedace. Both RNASeq libraries were sequenced to ∼37 M reads on the NovaSeq 6000 S4.

### Genome assembly—spikedace

Raw PacBio reads were assembled using Falcon Unzip 1.3.7 and produced a reference assembly of 1.18 Gb (Chin *et al*. 2016) as predicted. Because Illumina reads have a lower per-base error rate than PacBio, remaining frozen tissue was extracted and sequenced from the same spikedace individual to generate paired-end reads on the NovaSeq 6000 for error correction. Regions of high heterozygosity in eukaryotic genome assemblies often produce adjacent rather than collapsed copies of sequence, called haplotigs. Using the purge_dups v.1.2.3 pipeline, all haplotigs were identified and removed from further processing of the assembly (Guan *et al*. 2020). Pbmm2 from PacBio SMRT Tools was used to index the resulting purged assembly to generate a .mmi file and align raw PacBio reads. Polishing with raw PacBio reads was done using the variantCaller tool from PacBio SMRT Tools (GenomicConsensus v.2.3.3), specifying the arrow algorithm. Paired-end read data were used to further polish the assembly using a modified script written by Rohit Kolora (UC-Berkeley) for the Freebayes v.1.3.2 pipeline (Garrison *et al*. 2010). Dovetail Omni-C Library preps were made using tissue from the same flash-frozen tissue and sequenced on the NovaSeq 6000. Omni-C uses DNAse-based chemistry to fragment 3D-configured DNA in the cell prior to sequencing. Coverage based on Illumina, PacBio, or OmniC was determined using the BBMap v.38.76 reformat.sh script (Bushnell 2014).

Processing of Omni-C data was preceded by QC analysis using the Omni-C quality control script through Dovetail's Github repository (https://github.com/dovetail-genomics/). A library is considered to be acceptable if mapped nonduplicated pairs >1,000 bp is greater than 20% of the total mapped nonduplicated pairs and expected unique pairs at 300 M sequencing is at least ∼120 million. Scaffolding was then completed using the SALSA pipeline (Ghurye *et al*. 2017). From the Aiden Lab Github (https://github.com/aidenlab), we next generated a map of OmniC-C contacts with duplicates removed using the Juicer pipeline and input this along with our current assembly into the 3D-DNA pipeline to generate an OmniC contact map for downstream analysis (Durand, Shamim, *et al.* 2016 ; Dudchenko *et al*. 2017). Our assembly was modified based on the contact map to increase contiguity using Juicebox v.1.11.08 software and then exported with the 3D-DNA run-asm-pipeline-post-review.sh script (Durand, Robinson, *et al.* 2016). The modified assembly was assessed for quality by aligning paired-end Illumina reads to the new fasta file with bwa v.0.7.17-r1188 and sorting alignments with samtools v.1.8 (using htslib v.1.8) (Li and Durbin 2009 ; Li and Wysoker 2009). By plotting coverage across each chromosome, we located repeat-rich scaffolds or misassemblies to be designated as unplaced scaffolds and rearranged in Juicebox.

### Genome assembly—loach minnow

Initial processing of raw subreads for both cells generated for loach minnow began with generation of circular consensus reads command from (https://github.com/nlhepler/pbccs.git). Adapters were then removed from both bam files using HiFiAdapterFilt (Sim *et al.* 2022). Following concatenation of both trimmed bam files, we ran the assembler Hifiasm v. 0.15.1-r334 (Cheng *et al*. 2021). Scaffolding of the assembly was performed by Dovetail, Inc.

The input de novo assembly and Dovetail OmniC library reads were used as input data for HiRise, a software pipeline designed specifically for using proximity ligation data to scaffold genome assemblies (Putnam *et al*. 2016). Dovetail OmniC library sequences were aligned to the draft input assembly using bwa (https://github.com/lh3/bwa). The separations of Dovetail OmniC read pairs mapped within draft scaffolds were analyzed by HiRise to produce a likelihood model for genomic distance between read pairs, and the model was used to identify and break putative misjoins, to score prospective joins, and make joins above an optimal threshold determined during the scaffolding process (Putnam *et al*. 2016).

### Identification of repetitive regions and contamination

Repeat content needed to be masked as this can skew results of downstream population genetic analyses. Ancestral shared repeats were downloaded from Repbase using zebrafish (*Danio rerio*), a distantly related minnow species (Bao *et al*. 2015). RepeatModeler version 2.0.3 was then used to build a database of ancestral repeats in our current assembly (Flynn *et al*. 2020). The output fasta of Repeatmodeler was then input to Repeatmasker v.4.1.2 with the LTRStruct option enabled to identify transposable element families prior to softmasking (Tarailo-Graovac and Chen 2009).

Mitochondrial sequences were assembled using MitoZ v2.2 using 150 bp paired-end reads with an insert size parameter of 250 (Meng *et al*. 2019). Adapters were first trimmed with fastp v.0.23.2 and subset with extractfq due to memory constraints in MitoZ. We identified putative archaeal, bacterial, viral, and vector contamination by first downloading the RefSeq and UniVec database and creating a master fasta file for bacterial, archaeal, and viral sequences. Using Minimap2 (Li 2018), we mapped each database fasta and the mitochondrial genome against the reference genome assembly. We used a custom bedtools v.2.28.0 script to calculate the percent of summed contaminant and mitochondrial alignments represented on each genomic contig (Quinlan and Hall 2010). Bedtools maskfasta was then used to softmask sequences containing putative contamination.

Finally, raw PacBio subreads were converted into fastq format using bedtools bamtofastq and converted into fasta format with seqtk v.1.3-r106 (https://github.com/lh3/seqtk.git). Subreads in fasta format were then used to close gaps in the assembly using TGS-GapCloser v1.1.1 (Xu *et al*. 2020).

### Completeness

Quality of the assembly was assessed using N50 values and BUSCO scores. The assembly size was determined through QUAST v.5.0.2 along with assembly metrics including N50 and contig size distribution (Gurevich *et al*. 2013). Genome completeness was assessed with the actinopterygii_odb10 database in BUSCO v.4.1.4 (Seppey *et al*. 2019). Quast and BUSCO were run for both spikedace and the current zebrafish assembly (GRCz11) for comparative purposes. A genome-genome alignment with the zebrafish assembly GRCz11 was conducted in the Dgenies portal to identify homologous chromosomal identity using the Minimap2 algorithm (Cabanettes and Klopp 2018). Completeness was additionally assessed using OrthoVenn2, using killifish (*Fundulus heteroclitus*), guppy (*Poecilia reticulata*), and zebrafish (*D. rerio*) for orthogroup comparison (Xu *et al*. 2019) (Supplementary Fig. 1). We counted the sizes of remaining gaps in the assembly using a custom bash script and was visualized in R using ggplot2 (Supplementary Fig. 2) (Wickham 2011).

### Annotation—spikedace

Genome annotation was completed using RNASeq data generated for each species (spikedace N = 3, loach minnow N = 2). All genome annotation for spikedace was completed by Dovetail Inc. Repeat families found in the genome assemblies of spikedace were identified de novo and classified using the software package RepeatModeler (version 2.0.1) (Flynn *et al*. 2020). RepeatModeler depends on the programs RECON (version 1.08) and RepeatScout (version 1.0.6) for de novo identification of repeats within the genome (Bao and Eddy 2002; Price *et al*. 2005). The custom repeat library obtained from RepeatModeler was used to discover, identify, and mask the repeats in the assembly file using RepeatMasker (Version 4.1.0) (Tarailo-Graovac and Chen 2009). Coding sequences from zebrafish, *Onychostoma macrolepis*, and fathead minnow (*Pimephales promelas*) were used to train the initial ab initio model for spikedace using the AUGUSTUS software (version 2.5.5) (Stanke and Morgenstern 2005). Six rounds of prediction optimization were done using AUGUSTUS. The same coding sequences were also used to train a separate ab initio model for spikedace using SNAP (version 2006-07-28) (Korf 2004). RNASeq reads were mapped onto the genome using the STAR aligner software (version 2.7) and intron hints generated with the bam2hints tools within the AUGUSTUS software (Stanke and Morgenstern 2005; Dobin *et al*. 2013). MAKER, SNAP, and AUGUSTUS (with intron–exon boundary hints provided from RNASeq) were then used to predict for genes in the repeat-masked reference genome (Korf 2004; Stanke and Morgenstern 2005; Cantarel *et al*. 2008). To help guide the prediction process, Swiss-Prot peptide sequences from the UniProt database were downloaded and used in conjunction with the protein sequences from zebrafish, *O. macrolepis*, and fathead minnow to generate peptide evidence in the Maker pipeline (UniProt Consortium 2021). Only genes that were predicted by both SNAP and AUGUSTUS softwares were retained in the final gene sets. To help assess the quality of the gene prediction, Annotation Edit Distance (AED) scores were generated for each of the predicted genes as part of the MAKER pipeline. Genes were further characterized for their putative function by performing a BLAST search of the peptide sequences against the UniProt database (Camacho *et al*. 2009). tRNA were predicted using the software tRNAscan-SE (version 2.05) (“No title”).

### Annotation—loach minnow

All cDNA sequence data were quality checked for both sequence quality and adapter content using fastqc v.0.11.7 (Andrews and Others 2010) and adapter content was trimmed using fastp v.0.20.0 (Chen *et al*. 2018). Annotation and gene prediction for loach minnow was performed using BRAKER2 (Brůna *et al*. 2021). The odb10 vertebrate protein database was first downloaded in fasta format from OrthoDB (Kriventseva *et al*. 2019) and combined with the protein fasta from the zebrafish (GRCz11) assembly. Braker was run separately using reference proteins and separately using RNASeq alignments, as recommended in the Braker2 manual. RNASeq data were aligned to the loach minnow reference assembly using STAR RNASeq aligner v.2.7.10a (Dobin *et al*. 2013). Alignment summary metrics were calculated using Picard v.2.23.0 CollectAlignmentSummaryMetrics and then plotted counts of aligned reads using ggplot2 (Wickham 2011; Institute 2019) (Supplementary Fig. 3). The output binary alignment file was then sorted and indexed prior to inclusion in the second run of Braker2. GTF files from both runs were then combined using TSEBRA (Gabriel *et al*. 2021) to combine the results of both runs. All software and versions for major steps in the genome assembly process are listed in Supplementary Table 1.

Protein fasta files for both assemblies first had all asterisks and gaps removed and were then input to Interproscan v.5.52-86.0 and run using all available analyses (Jones *et al*. 2014). We then ran the funannotate annotate function v.1.8.13 (Li and Wang 2021) using the vertebrate BUSCO dataset (Seppey *et al*. 2019), and merged output from the EGGNOG web interface (Cantalapiedra *et al*. 2021) along with previous Interproscan results in XML format into a final gff3 file. Lastly, both AUGUSTUS and BRAKER2 tend to over predict the presence of single exon genes (SEGs) during annotation. To ameliorate this issue, we only retained SEGs in which the resulting protein was supported by hits in the Interproscan database. We first identified transcripts with single exons by parsing the output from *genestats.sh* (https://github.com/darencard/GenomeAnnotation). A bash script was used to search for SEG transcripts which had annotation hits using Interproscan and SEGs without hits were then removed using *agat_sp_filter_feature_from_kill_list.pl* (https://github.com/NBISweden/AGAT).

### Comparative genomics

Final annotated assembles of spikedace and loach minnow were used in comparative genomic analyses. Specifically, we were interested in the identification of syntenic regions and structural rearrangements, gene family expansions, and genes under positive selection. The following taxa were used in the various analyses outlined below: sunbleak (*Leucaspius delineatus*; GenBank: GCA_023718395.1), common carp (*Cyprinus carpio*; GenBank: GCA_018340385.1), goldfish (*Carassius auratus*; GenBank: GCA_003368295.1), zebrafish (GenBank: GCA_000002035.4), and fathead minnow (GenBank: GCA_016745375.1). Sunbleak was chosen for comparison rather than the more closely related fathead minnow as the sunbleak genome was chromosome level and could provide unique genome rearrangements to Cyprinids unique to North America.

Chromosomes from spikedace and loach minnow assemblies were aligned to the sunbleak reference sequence using minimap2 v2.24 with default parameters (-ax -asm5) (Li 2018). Syntenic regions and structural rearrangements were identified using Syri v1.6.3 (Goel *et al*. 2019) and visualized with plotsr v0.5.4 (Goel and Schneeberger 2022).

We identified gene family expansions using Orthofinder (v 2.5.4) (Emms and Kelly 2019). Briefly, the longest isoform of each protein was extracted from the protein annotated fasta files and used as input for each of the following species: common carp, goldfish, zebrafish, spikedace, fathead minnow, and loach minnow. Default parameters were used and the species tree recovered followed the branching pattern exhibited in a phylogeny with more complete taxonomic sampling (Schönhuth *et al*. 2018). Output from Orthofinder (Orthogroups.GeneCount.tsv) was parsed to find the 5 numerically largest orthogroups within both spikedace and loach minnow that retained at least a single ortholog in zebrafish. We also searched for gene family expansions present in both genomes. To find expansions of comparable size, we calculated the absolute difference in gene counts per orthogroup between spikedace and loach minnow and retained those with a difference of less than 5. We then summed gene counts within remaining orthogroups for spikedace and loach minnow and isolated orthogroups where the sum was at least 10 times greater than the number of orthologs observed in zebrafish. Zebrafish orthologs were used to explore functional enrichment associated with the expanded gene families identified in spikedace and loach minnow. We report enrichment for gene ontology terms and protein domains using StringDB (Szklarczyk *et al*. 2021). Reported *P*-values were corrected using a 5% false discovery rate.

Lastly, we wanted to test whether any genes showed patterns of episodic selection on the branches leading to loach minnow and spikedace. Because loach minnow (Pogonichthyinae) and spikedace (Plagopterinae) are phylogenetically distant within Cypriniformes (Schönhuth *et al*. 2018), comparative genomics can shed light on convergent adaptation to extreme environments such as deserts. Four high-quality cyprinid reference genomes were downloaded from NCBI for comparison: common carp, goldfish, fathead minnow, and zebrafish. Annotated proteins were also downloaded and included with annotated proteins for spikedace and loach minnow as input for Orthofinder (v.2.5.4) (Emms and Kelly 2019). We ran Orthofinder using mafft and fastree in order to improve Orthofinder's alignment accuracy using the -M msa option and identified single-copy orthologs across all 6 assemblies. Coding sequence (CDS) could differ slightly from the protein fasta sequences associated with each assembly, depending on whether annotation was RNA-based and came from a different individual or DNA strand, creating problems for downstream alignment steps. To remedy this, we extracted CDS sequences from each genome using gffread (v.0.12.7), identified each gene based on protein ID, and extracted nucleotide sequences based on the gff file associated with each genome (Pertea and Pertea 2020).

Each CDS sequence represented as a single-copy ortholog across species was then translated into protein sequence using EMBOSS transeq (Rice *et al*. 2000) and aligned using Clustal Omega (v.1.2.4) (Sievers and Higgins 2014). From each protein alignment, proteins were converted back into coding nucleotide alignments using pal2nal (v.14) (Suyama *et al*. 2006). The concatenated fasta file containing CDS from each genome, using protein ID as the headers, was then indexed with cdbfasta (Pertea). Then each respective gene tree identified in Orthofinder was appended with protein labels using the cdbfasta index.

We implemented a test for gene-wide episodic selection in HyPhy (Pond and Muse 2005) using the BUSTED algorithm (Murrell *et al*. 2015). Briefly, the BUSTED algorithm has the ability to identify increases in the rate of nonsynonymous substitutions on a set of prespecified foreground labels relative to the background and has increased power over the branch-site model in PAML (Murrell *et al*. 2015). We ran the HyPhy BUSTED algorithm on all genes in parallel by specifying the gene tree and nucleotide alignment for each single-copy ortholog. Each gene tree was converted into newick format and annotated with foreground labels on proteins that were identified in spikedace or loach minnow. All protein names from genes identified as showing signatures of episodic selection in foreground labels with a *P*-value of <0.05 were then extracted and input to the StringDB (Szklarczyk *et al*. 2021). We looked for terms that were significantly enriched across StringDB databases for zebrafish and reported those with *P*-values of <0.05 corrected with for false discovery rate then sorted results by strength of the enrichment.

## Results and discussion

### Genome assembly and annotation

Summary statistics for spikedace and loach minnow genome assemblies were of similar quality to the model zebrafish genome assembly. For example, N50 values were similar for the 3 assemblies, with values for loach minnow and spikedace slightly below that of zebrafish. The contiguity of the loach minnow and spikedace assemblies is likely in part an artifact of smaller chromosome and genome sizes in both species, which is indicated by the size of the largest contig in each assembly (Table 1). We additionally observed an improvement over the zebrafish assembly because of a reduction in the overall number of contigs and gaps per 100 kb.

**Table 1.** Summary statistics output from Quast comparing genome assemblies for spikedace (*Meda fulgida*), loach minnow (*Tiaroga cobitis*), and zebrafish (*Danio rerio*; GRCz11).

Assembly statistics	Spikedace	Loach minnow	Zebrafish
# contigs (≥0 bp)	83	550	1,923
# contigs (≥1,000 bp)	83	550	1,922
# contigs (≥5,000 bp)	72	550	1,824
# contigs (≥10,000 bp)	70	550	1,727
# contigs (≥25,000 bp)	68	499	1,281
# contigs (≥50,000 bp)	64	283	1,091
Total length (≥0 bp)	882,120,208	1,320,521,697	1,679,203,469
Total length (≥1,000 bp)	882,120,208	1,320,521,697	1,679,202,819
Total length (≥5,000 bp)	882,100,741	1,320,521,697	1,678,981,660
Total length (≥10,000 bp)	882,090,741	1,320,521,697	1,678,178,846
Total length (≥25,000 bp)	882,054,741	1,319,423,137	1,671,157,076
Total length (≥50,000 bp)	881,926,514	1,311,861,986	1,664,545,481
# contigs	83	550	1,923
Largest contig	52,739,976	67,399,795	78,093,715
Total length	882,120,208	1,320,521,697	1,679,203,469
GC (%)	39.33	40.28	36.60
N50	34,805,954	48,679,909	52,186,027
N75	32,365,017	42,382,842	42,172,926
L50	11	13	14
L75	18	20	23
# N's per 100 kbp	8.14	3.02	279.51

We identified slight improvements in completeness of loach minnow and spikedace assemblies compared with that of zebrafish when BUSCO metrics were compared directly with the Actinopterygii BUSCO database. In summary, BUSCO scores showed a ∼1–2% improvement in percent of complete BUSCO proportions with ∼97% for spikedace and ∼98% for loach minnow relative to ∼96% for zebrafish (Table 2). Furthermore, we report lower numbers of duplicated, fragmented, and missing BUSCOs in both spikedace and loach minnow relative to the zebrafish.

**Table 2.** BUSCO scores comparing genome assemblies for spikedace (*Meda fulgida*), loach minnow (*Tiaroga cobitis*), and zebrafish (*Danio rerio*; GRCz11).

	Spikedace		Loach minnow		Zebrafish	
BUSCO metric	Percent	Total	Percent	Total	Percent	Total
Complete	96.90%	3,529	97.70%	3,556	95.90%	3,494
Single copy	95.60%	3,481	94.50%	3,441	78.50%	2,859
Duplicated	1.30%	48	3.20%	115	17.40%	635
Fragmented	1%	35	0.90%	32	1.70%	61
Missing	2.10%	76	1.40%	52	2.40%	85
Total	3,640					

Bacterial contamination was low for both assemblies, in each case found on 8 scaffolds representing 0–0.006% of chromosome length or up to 2% of unplaced scaffold length (Supplementary Table 2). Putative bacterial contamination was softmasked. Mitochondrial contamination, in contrast, was only found in the Hifiasm assembly for loach minnow. Mitochondrial contamination was found on 6 scaffolds representing 40–90% of the scaffold length, so each of these scaffolds were removed from the final assembly. Because these alignments were roughly the size of the mitochondrial genome (16 kb), they were not considered to be mitonuclear genes. We did not detect any evidence for viral contamination.

Repeat content was similar between the 2 assemblies with around 48% of the genome masked in loach minnow (Supplementary Table 3) and ∼60% in spikedace (Supplementary Table 4). In both assemblies, retroelements made up 13–15%, DNA transposons were ∼17–20%, rolling-circles were ∼1–3%, and ∼10–12% was unclassified. In both, subsets of repeat types were additionally conserved where 5% of retroelements were long interspersed nuclear elements (LINEs): 4% of types L2, CR1, or Rex elements. A total of 10% of retroelements were LTR elements, which were mostly Gypsy or DIRS1 elements (∼7%). DNA transposons were mostly made up of hobo-activators at 4–5% or Tc1-IS630-Pogo elements (∼1–2%). Repeat classes and families by percentage of the total genome size were similar between both genomes. Excluding unknown repeats, the largest class/family in loach minnow was simple repeats, while a large proportion of LTR/DIRS repeats were present in both spikedace and loach minnow, being the largest class/family in spikedace (Supplementary Fig. 4).

Gap size distribution varied between assemblies with ∼70 kb of gapped sequence in spikedace and 40-kb sequence in loach minnow. Mean gap size was around 400 bp lower in loach minnow (∼100 bp) than in spikedace (∼500 bp), likely as a result of the lower error rate of circular consensus reads and the larger amount of sequence data for the loach minnow (Supplementary Table 5). The majority of gaps in the loach minnow assembly were small with a minimum of 1, while those of the spikedace were larger with a minimum of 50, although the maximum for both was similar (∼1–1.4 kb).

Following annotation, a total of 282 and 11,419 SEGs were filtered from spikedace and loach minnow gff files, respectively. Summaries presented here are based on filtered results. We annotated 38,671 genes in spikedace with an average length of 7,432.71 bp covering approximately 32.58% of total genome length. A total of 278,879 exons were identified with a mean of 7.25 exons and 6.25 introns per mRNA transcript. We annotated 37,413 genes within the loach minnow genome with a mean length of 10,252.93 bp that covered 29.04% of total genome length. A total of 281,170 exons were identified with an average of 7.01 exons and 6.02 introns per mRNA transcript. The distributions of mRNA transcripts lengths, number of exons per transcript, number of introns per transcript, and CDS lengths are presented for both genomes in Supplementary Fig. 5a–d.

### Comparative genomics

By comparing spikedace and loach minnow assemblies against the sunbleak reference, we found 574.4 and 655.2 Mb of syntenic regions, corresponding to 66.2 and 54.4% of our assemblies, respectively. In addition, we found 62.4 and 87.9 Mb of structural rearrangements, corresponding to 7.2 and 7.3% of the spikedace and loach minnow assemblies, respectively (Supplementary Fig. 6). Rearrangements included 57.2–75.6 Mb (114–123) of inversions, 2.7–9.1 Mb (1896–5483) of translocations, and 2.5–3.2 Mb (1962–2243) of duplications. (Supplementary Table 6). Of these identified rearrangements, 17.8 Mb of inversions, 90 kb of translocations, and 4.0 Mb of duplications were shared between spikedace and loach minnow.

Among expanded gene families in spikedace, we found no evidence for significantly enriched GO terms, but we did recover enriched protein domains related to leucine rich repeat N-terminal (SM00013; *P* = 0.0184), leucine rich repeats subfamily (SM00369; *P* = 0.0184), Transposase (PF01498; *P* = 0.00021) and Homeodomain-like (PF13384; *P* = 0.00021) in the 2 largest orthogroup (Supplementary Table 7). A single gene family in loach minnow was enriched for molecular function and cellular component GO terms and 3 protein domains (Supplementary Table 7). Enriched GO terms and protein domains included: serin-type endopeptidase inhibitor activity (GO:0004867; *P* = 2.77e^−14^), extracellular space (GO:0005615; *P* = 1.19e^−06^), A-macroglobulin receptor (SM01361; *P* = 1.17e^−08^), Alpha 2-macroglobulin family (SM01360; *P* = 2.78e^−15^), and Alpha 2-macroglobulin (SM01359; *P* = 2.78e^−15^). There was no evidence for functional enrichment in gene family expansions present in both spikedace and loach minnow. However, there was a propensity for increased gene numbers in spikedace and loach minnow to be associated with transposable element, retrotransposon, and ion binding orthologs in zebrafish.

From the Orthofinder output, we identified a total of 1,465 genes with single-copy orthologs between all 6 genome assemblies. From this total, 371 genes showed evidence of episodic selection (*P* < 0.05) (Supplementary Table 8). In total, 11 terms were enriched using the StringDB Biological Process database, most of which were related to cellular metabolic processes (Supplementary Table 9).

The high-quality reference genomes for spikedace and loach minnow presented here provide new insight as how chromosome evolution has shaped these 2 endemic, monotypic lineages. Comparisons with other temperate cyprinids suggest that a series of structural rearrangements are shared between loach minnow and spikedace, most of which were inversions, that could play a role in speciation, adaptation, or regulation of gene expression. With respect to gene content, expanded gene families tended to be associated with repetitive and transposable elements, which are structural classes hypothesized to contribute evolutionary events among freshwater fishes (Shao *et al*. 2019). We also found expanded gene families associated with ion binding that is potentially related to adaptation to higher conductivity that is characteristic of arid-land streams (Griffith 2014) and a gene related to low oxygen and high temperatures in aquatic environments (SOD3a) (Sun *et al*. 2020). Moreover, we found evidence of episodic positive selection that has occurred independently in loach minnow and spikedace relative to temperate cyprinids, with the largest enrichment in gene function for terms related to cellular metabolic processes. A large number of genes found showing a unique signal of episodic positive selection in loach minnow and spikedace have known associations with oxidative stress (p67phox, urate oxidase, ATM, PEX6p, SQSTM1, and TRIM37) (Peroxisomes), folate deficiency (MTHFD1, ABCC1, FOLR1, ALDH7A1, and Lrp2) (Au *et al*. 2017) (which affects the production of red blood cells), development of muscle (tspan34, popdc2, top2a, and tmod1) (Louie *et al*. 2017), and DNA repair (XRCC3, XRCC1, FANCG, NTHL1, and ATM) (Song *et al*. 2012), all of which may be related to the unique ecology of these 2 species.

Genomic resources presented in this paper help improve our understanding of how the diversity of freshwater fishes is generated from a genomic view, and aids in biodiversity conservation. The consistent decline in native desert fish populations due to anthropogenic activities has resulted in the increased isolation of many species (Meffe and Vrijenhoek 1988) and concomitantly increased risk of extirpation and extinction. Preserving the biodiversity of freshwater fishes is essential for preserving both the ecosystem services they provide in their native habitat and the scientific value they provide in understanding diversity, particularly in the face of a changing planet. With reference genomes currently being generated at an unprecedented pace, genetic assessments of vulnerability in at-risk populations have become increasingly feasible to better inform management decisions (Formenti *et al*. 2022). The conservation of native fishes in the Gila River basin has focused primarily on the prioritization of populations inhabiting the least disturbed habitat (Hickerson *et al*. 2022). These new reference assemblies provide the additional benefit of incorporating estimates of standing genetic diversity and inbreeding on a per-population basis for management decisions, including inferences of past and future predictions of population risk.

## Supplementary Material

jkad157_Supplementary_Data

## Data Availability

All metadata associated with this assembly can be found in the main text. Raw sequence data and the genome fasta files were deposited to GenBank. The final DNA sequence assemblies and raw sequence data can be found under the following bioproject PRJNA947235. The annotation file in gff3 format can be found on our Github repository at https://github.com/nicolasalexandre21/Minnow. Supplemental material available at G3 online .
