# Supplementary material for: Chromosome-level reference genomes of two imperiled desert fishes: spikedace (Meda fulgida) and loach minnow (Tiaroga cobitis)
Source: G3 (Bethesda). 2023 Jul 19;13(10):jkad157. doi: 10.1093/g3journal/jkad157 (PMC10542311; doi:10.1093/g3journal/jkad157)
Supplement: jkad157_Supplementary_Data [file jkad157_supplementary_data.docx]

**Figures and Tables**

| **Spikedace** | | | |
| --- | --- | --- | --- |
| **Assembly Step** | **Software** | **Version** |  |
| Genome Assembler | Falcon | 1.3.7 |  |
| Haplotig Removal | PurgeDups | 1.2.3 |  |
| Polishing | FreeBayes | 1.3.2 |  |
| Broad-scale Scaffolding | SALSA | 2.0 |  |
| Fine-scale Scaffolding | Juicebox | 1.11.08 |  |
| **Loach Minnow** | | | |
| **Assembly Step** | **Software** | **Version** |  |
| Consensus Read Generation | Pbccs | 1.0 |  |
| Adapter Removal | HiFiAdapterFilt | 2.0.0 |  |
| Genome Assembler | Hifiasm | 0.15.1-r334 |  |
| **Both Genomes** | | | |
| **Assembly Step** | **Software** | **Version** |  |
| Repeat Modeling | RepeatModeler | 2.0.3 |  |
| Repeat Masking | RepeatMasker | 4.1.2 |  |
| Mitochondrial Assembly | Mitoz | 2.2 |  |
| Gap Closing | TGS-GapCloser | 1.1.1 |  |

Table S1. All installed software packages used for both genomes along with the relevant versions.

| **Loach Minnow** | | | | |
| --- | --- | --- | --- | --- |
| **Scaffold** | **Alignment Size** | **Scaffold Size (bp)** | **Percent of Scaffold** | **Contamination Type** |
| Chr12 | 50 | 42382842 | 0.000117972 | Bacterial |
| Chr20 | 61 | 45255464 | 0.00013479 | Bacterial |
| Chr21 | 129 | 54984250 | 0.000234613 | Bacterial |
| Chr2 | 134 | 54139342 | 0.000247509 | Bacterial |
| Chr24 | 144 | 33849805 | 0.000425409 | Bacterial |
| Chr8 | 550 | 48535966 | 0.00113318 | Bacterial |
| Chr6 | 1352 | 48741992 | 0.00277379 | Bacterial |
| Sca222 | 1417 | 68203 | 2.07762 | Bacterial |
| Sca329 | 16663 | 40646 | 40.9954 | Mitochondrial |
| Sca461 | 16652 | 28961 | 57.498 | Mitochondrial |
| Sca509 | 16217 | 23547 | 68.8708 | Mitochondrial |
| Sca515 | 16276 | 23134 | 70.3553 | Mitochondrial |
| Sca522 | 16428 | 22496 | 73.0263 | Mitochondrial |
| Sca544 | 16354 | 18204 | 89.8374 | Mitochondrial |
| **Spikedace** | | | | |
| **Scaffold** | **Alignment Size** | **Scaffold Size (bp)** | **Percent of Scaffold** | **Contamination Type** |
| Chr1 | 95 | 42060603 | 0.000225865 | Bacterial |
| Chr10 | 79 | 25211462 | 0.00031335 | Bacterial |
| Chr20 | 108 | 34307314 | 0.000314802 | Bacterial |
| Chr18 | 192 | 38590863 | 0.000497527 | Bacterial |
| Chr17 | 319 | 34433719 | 0.000926418 | Bacterial |
| Chr19 | 615 | 33588571 | 0.00183098 | Bacterial |
| Chr2 | 920 | 38444313 | 0.00239307 | Bacterial |
| Chr9 | 2379 | 38753325 | 0.00613883 | Bacterial |

Table S2. Candidate contigs containing mitochondrial or bacterial contamination along with alignment length and percent of reference scaffold covered.

| **Loach Minnow** |
| --- |
| sequences:            83 |
| total length:  874550422 bp  (873927538 bp excl N/X-runs) |
| GC level:         39.31 % |
| bases masked:  427017800 bp ( 48.83 %) |
| **Element # Elements Length Percent of Sequence** |
| Retroelements       247960    136521393 bp   15.61 % |
| SINEs:             9768      1792311 bp    0.20 % |
| Penelope           1491       633603 bp    0.07 % |
| LINEs:           101005     44623299 bp    5.10 % |
| CRE/SLACS            0            0 bp    0.00 % |
| L2/CR1/Rex      82439     36081057 bp    4.13 % |
| R1/LOA/Jockey    2706      1042298 bp    0.12 % |
| R2/R4/NeSL       1436       986430 bp    0.11 % |
| RTE/Bov-B        4994      1272005 bp    0.15 % |
| L1/CIN4          2017       808104 bp    0.09 % |
| LTR elements:    137187     90105783 bp   10.30 % |
| BEL/Pao          9980      7164017 bp    0.82 % |
| Ty1/Copia         388       230657 bp    0.03 % |
| Gypsy/DIRS1     80310     61817140 bp    7.07 % |
| Retroviral    10399      9621013 bp    1.10 % |
| DNA transposons     775326    150901645 bp   17.25 % |
| hobo-Activator   198812     36709646 bp    4.20 % |
| Tc1-IS630-Pogo    59183     23855921 bp    2.73 % |
| En-Spm                0            0 bp    0.00 % |
| MuDR-IS905            0            0 bp    0.00 % |
| PiggyBac          13169      2512067 bp    0.29 % |
| Tourist/Harbinger 57946     12282742 bp    1.40 % |
| Other (Mirage,     3986      1158111 bp    0.13 % |
| P-element, Transib) |
| Rolling-circles      27107      9838707 bp    1.13 % |
| Unclassified:       539590     92657307 bp   10.59 % |
| Total interspersed repeats:   380080345 bp   43.46 % |
| Small RNA:           11024      1843802 bp    0.21 % |
| Satellites:          55976      9466710 bp    1.08 % |
| Simple repeats:     355637     24082952 bp    2.75 % |
| Low complexity:      31838      1859848 bp    0.21 % |

Table S3. Output of RepeatMasker showing the total number of repeats by repeat class for Loach Minnow.

| **Spikedace** |
| --- |
| sequences:           550 |
| total length: 1320517157 bp  (1320479757 bp excl N/X-runs) |
| GC level:         40.28 % |
| bases masked:  814399811 bp ( 61.67 %) |
| **Element # Elements Length Percent of Sequence** |
| Retroelements       275690    181976611 bp   13.78 % |
| SINEs:            27898      4983484 bp    0.38 % |
| Penelope           1649       641731 bp    0.05 % |
| LINEs:           124716     65317798 bp    4.95 % |
| CRE/SLACS            0            0 bp    0.00 % |
| L2/CR1/Rex      96069     47367091 bp    3.59 % |
| R1/LOA/Jockey    4635      2037702 bp    0.15 % |
| R2/R4/NeSL       4980      3428868 bp    0.26 % |
| RTE/Bov-B        4107      2510389 bp    0.19 % |
| L1/CIN4          4979      2010694 bp    0.15 % |
| LTR elements:    123076    111675329 bp    8.46 % |
| BEL/Pao          3917      4283743 bp    0.32 % |
| Ty1/Copia           0            0 bp    0.00 % |
| Gypsy/DIRS1     88657     87000917 bp    6.59 % |
| Retroviral    15951     15455342 bp    1.17 % |
| DNA transposons     1120662    263553081 bp   19.96 % |
| hobo-Activator   331539     75437622 bp    5.71 % |
| Tc1-IS630-Pogo    70294     22088326 bp    1.67 % |
| En-Spm                0            0 bp    0.00 % |
| MuDR-IS905            0            0 bp    0.00 % |
| PiggyBac          41580     17690755 bp    1.34 % |
| Tourist/Harbinger 82854     19931055 bp    1.51 % |
| Other (Mirage,     8807      2159341 bp    0.16 % |
| P-element, Transib) |
| Rolling-circles      42240     43900223 bp    3.32 % |
| Unclassified:       784270    168541761 bp   12.76 % |
| Total interspersed repeats:   614071453 bp   46.50 % |
| Small RNA:           22243      8565768 bp    0.65 % |
| Satellites:          63281     14020729 bp    1.06 % |
| Simple repeats:     430003    129610038 bp    9.82 % |
| Low complexity:      35454      4645944 bp    0.35 % |

Table S4. Output of RepeatMasker showing the total number of repeats by repeat class for Spikedace.

| **Species** | **Minimum** | **1st Qtl.** | **Median** | **Mean** | **3rd Qtl.** | **Maximum** | **Total Gap Size** |
| --- | --- | --- | --- | --- | --- | --- | --- |
| **Spikedace** | 1 | 500 | 500 | 485 | 500 | 1023 | 71784 |
| **Loach Minnow** | 50 | 100 | 100 | 106.8 | 100 | 1417 | 39837 |

Table S5. Summary statistics for the distribution of gap sizes in each genome assembly.

|  | **Spikedace** | | **Loach Minnow** | |
| --- | --- | --- | --- | --- |
| **Variation** | **Count** | **Length (bp)** | **Count** | **Length (bp)** |
| **SYN** | 1,069 | 574,360,328 | 1,373 | 655,211,180 |
| **INV** | 123 | 57,190,210 | 114 | 75,629,599 |
| **TRN** | 1,896 | 2,749,186 | 5,483 | 9,073,266 |
| **DUP-REF** | 371 | 733,669 | 667 | 1,636,077 |
| **DUP-QRY** | 1,962 | 2,468,029 | 2,243 | 3,153,663 |
| **UNQ-REF** | 3,410 | 419,715,976 | 7,421 | 476,079,246 |
| **UNQ-QRY** | 5,109 | 230,428,615 | 9,266 | 461,895,513 |
| **SNPs** | 1,333,088 | 1,333,088 | 1,626,712 | 1,626,712 |
| **INS** | 68,361 | 370,014 | 99,148 | 561,284 |
| **DEL** | 87,306 | 419,521 | 105,265 | 537,146 |
| **CPG** | 9 | 50,401 | 9 | 2,741 |
| **CPL** | 7 | 230,982 | 10 | 16,132 |

Table S6. Number and total length of syntenic, structural rearrangement, and local variation regions. SYN: syntenic region; INV: inversion; TRN: translocation; DUP-REF: duplication found in the reference but not query genome; DUP-QRY: duplication found in the query but not reference genome; UNQ-REF: unaligned regions specific to the reference; UNQ-QRY: unaligned regions specific to the query; SNPs: single nucleotide polymorphisms; INS: insertions; DEL: deletions; CPG: copy gain variation; CPL: copy loss variation.

| **Spikedace** |  |  |  |  |  |
| --- | --- | --- | --- | --- | --- |
| ***de novo* Counts** | **Zebrafish Counts** | **Gene**  **Symbols** | **Sequence IDs** | **Description/Summary** | **Enriched**  **Terms** |
| 127 | 2 | si:dkey-182i3.11  lrfn4b | XP_021322095.1  XP_021333806.1 | leucine-rich repeat-containing protein 15-like  leucine rich repeat and fibronectin type III domain containing 4b | SM00013  SM00369 |
| 123 | 4 | LOC110438296  si:ch211-276i12.9  zgc:194285 | XP_021325357.1  XP_009289536.1  XP_005169673.1  NP_001129452.1 | muscle M-line assembly protein unc-89-like  IS630 family transposase; Predicted to have DNA binding activity.  IS630 family transposase; Predicted to have DNA binding activity. Predicted to be involved in DNA integration and transposition, DNA-mediated | PF01498  PF13384 |
| 101 | 2 | LOC108184914  LOC110438106 | XP_021331573.1  XP_021323532.1 | uncharacterized protein  uncharacterized protein | NA |
| 74 | 1 | lrrc74b | XP_001344236.1 | leucine rich repeat containing 74B; orthologous to human LRRC74B | NA |
| 64 | 1 | LOC108182677 | XP_021326638.1 | uncharacterized protein | NA |
| **Loach Minnow** |  |  |  |  |  |
| 42 | 1 | LOC103910304 | XP_021330371.1 | uncharacterized protein | NA |
| 42 | 1 | LOC110438779 | XP_021327606.1 | uncharacterized protein | NA |
| 37 | 11 | LOC108190122  LOC110440162 | XP_017211834.1  XP_017211835.1  XP_017211836.1  XP_021327357.1  XP_021327358.1  XP_021332313.1  XP_021332314.1  XP_021332315.1  XP_021332316.1  XP_021332317.1  XP_021336140.1 | retrovirus-related Pol polyprotein from transposon 412  uncharacterized protein | NA |
| 35 | 8 | zgc:165518  zgc:171445  zgc:171446  si:dkey-46g23.5  zgc:165453  LOC103909927  zgc:171426  si:dkey-46g23.2 | NP_001093623.2  NP_001107101.1  NP_001108028.1  NP_001188334.2  XP_009297326.1  XP_009296557.2  XP_017210396.1  XP_021327775.1 | Predicted to enable serine-type endopeptidase inhibitor activity. Predicted to act upstream of or within negative regulation of peptidase activity. Predicted to be located in extracellular space  “ ”  “ ”  “ ”  “ ”  pregnancy zone protein-like  Predicted to be located in extracellular space  alpha-2-macroglobulin-like protein 1 | GO:0004867  GO:0005615  SM01361  SM01360  SM01359 |
| 35 | 1 | sod3a | NP_001092706.1 | superoxide dismutase 3, extracellular a; Predicted to enable copper ion binding activity and superoxide dismutase activity. Predicted to be involved in removal of superoxide radicals. Predicted to act upstream of or within superoxide metabolic process |  |
| **Both Species** |  |  |  |  |  |
| 35 (16;19) | 1 | LOC101883290 | XP_005161423.1 | uncharacterized protein | NA |
| 34 (18;16) | 1 | si:ch211-79h18.2 | XP_009303290.1 | uncharacterized protein | NA |
| 29 (13;16) | 1 | LOC110439882 | XP_021333025.1 | uncharacterized protein | NA |
| 20 (8;12) | 2 | LOC103909745 | XP_021325967.1  XP_021325968.1 | G2/M phase-specific E3 ubiquitin-protein ligase-like | NA |
| 16 (7;9) | 1 | si:dkey-56d12.4 | NP_001103508.1 | Predicted to enable DNA binding activity and metal ion binding activity. | NA |
| 16 (9;7) | 1 | LOC110437757 | XP_003200989.3 | piggyBac transposable element-derived protein 3-like | NA |
| 16 (9:7) | 1 | phax | NP_001003995.1 | phosphorylated adaptor for RNA export; Predicted to enable RNA binding activity. Predicted to be involved in snRNA export from nucleus. Predicted to act upstream of or within protein transport. Predicted to be located in cytoplasm. Predicted to be active in nucleus. | NA |

Table S7. Results of gene family expansion analysis for the largest gene families with at least a single ortholog in zebrafish. We provide the number of genes in each orthogroup for Spikedace and Loach Minnow (de novo counts), number of Zebrafish orthologues, corresponding zebrafish gene symbols and sequence IDs within gene families. We also provided enriched GO terms and protein domains. For gene family expansions in both species we provide the sum of Spikedace and Loach Minnow and individual counts in parentheses (Spikedace:LoachMinnow).

| **Node** | **Identifier** |
| --- | --- |
| ABCC1 | 7955.ENSDARP00000078094 |
| ACOT12 | 7955.ENSDARP00000071202 |
| ANKLE1 | 7955.ENSDARP00000105049 |
| APH1A | 7955.ENSDARP00000069062 |
| C10H5orf63 | 7955.ENSDARP00000116545 |
| C16H8orf76 | 7955.ENSDARP00000103375 |
| CFAP99 | 7955.ENSDARP00000127983 |
| CLCC1 | 7955.ENSDARP00000107966 |
| DEPDC4 | 7955.ENSDARP00000108905 |
| ENSDARP00000073899 | 7955.ENSDARP00000073899 |
| FAM131A | 7955.ENSDARP00000100748 |
| FOLR1 | 7955.ENSDARP00000098284 |
| GPN1 | 7955.ENSDARP00000121489 |
| HSPB11 | 7955.ENSDARP00000088395 |
| LMTK3 | 7955.ENSDARP00000116726 |
| LOC100332753 | 7955.ENSDARP00000105584 |
| LOC557824 | 7955.ENSDARP00000128969 |
| LOC557848 | 7955.ENSDARP00000128352 |
| LOC557854 | 7955.ENSDARP00000059203 |
| LOC559514 | 7955.ENSDARP00000129614 |
| LOC559575 | 7955.ENSDARP00000128169 |
| LOC559650 | 7955.ENSDARP00000128742 |
| LOC560618 | 7955.ENSDARP00000104347 |
| LOC566378 | 7955.ENSDARP00000109897 |
| LOC566523 | 7955.ENSDARP00000129871 |
| LOC569419 | 7955.ENSDARP00000111624 |
| LOC570613 | 7955.ENSDARP00000069904 |
| LOC571740 | 7955.ENSDARP00000099540 |
| LOC571865 | 7955.ENSDARP00000098057 |
| LOC793673 | 7955.ENSDARP00000128247 |
| LOC794198 | 7955.ENSDARP00000126914 |
| LOC794348 | 7955.ENSDARP00000104069 |
| LOC794519 | 7955.ENSDARP00000127542 |
| LOC799552 | 7955.ENSDARP00000099984 |
| MMP23B | 7955.ENSDARP00000063241 |
| NAALADL1 | 7955.ENSDARP00000073505 |
| NEK10 | 7955.ENSDARP00000109162 |
| NEK11 | 7955.ENSDARP00000115167 |
| NFX1 | 7955.ENSDARP00000101631 |
| OGFOD3 | 7955.ENSDARP00000100959 |
| PROSER1 | 7955.ENSDARP00000106355 |
| RMDN1 | 7955.ENSDARP00000101059 |
| RNF219 | 7955.ENSDARP00000107201 |
| SLC27A3 | 7955.ENSDARP00000067708 |
| SPAG5 | 7955.ENSDARP00000126902 |
| SVEP1 | 7955.ENSDARP00000071352 |
| SYDE1 | 7955.ENSDARP00000098809 |
| TONSL | 7955.ENSDARP00000096098 |
| UTP20 | 7955.ENSDARP00000038850 |
| VWA3A | 7955.ENSDARP00000107851 |
| ZSWIM2 | 7955.ENSDARP00000101455 |
| Zranb3 | 7955.ENSDARP00000109706 |
| aars2 | 7955.ENSDARP00000113382 |
| abcb10 | 7955.ENSDARP00000082438 |
| abcc6b.2 | 7955.ENSDARP00000118198 |
| abi2a | 7955.ENSDARP00000118040 |
| acads | 7955.ENSDARP00000014757 |
| acbd6 | 7955.ENSDARP00000057641 |
| acox3 | 7955.ENSDARP00000056744 |
| adhfe1 | 7955.ENSDARP00000098935 |
| aim1a | 7955.ENSDARP00000115118 |
| aldh7a1 | 7955.ENSDARP00000108190 |
| alg11 | 7955.ENSDARP00000049513 |
| ankrd28b | 7955.ENSDARP00000020838 |
| ankrd49 | 7955.ENSDARP00000030673 |
| aqr | 7955.ENSDARP00000110866 |
| asb1 | 7955.ENSDARP00000030625 |
| asxl2 | 7955.ENSDARP00000100263 |
| asz1 | 7955.ENSDARP00000021759 |
| atm | 7955.ENSDARP00000080608 |
| bag3 | 7955.ENSDARP00000057688 |
| brms1la | 7955.ENSDARP00000008214 |
| c9 | 7955.ENSDARP00000121323 |
| cars2 | 7955.ENSDARP00000103031 |
| ccdc104 | 7955.ENSDARP00000093287 |
| cdk12 | 7955.ENSDARP00000087834 |
| chd1l | 7955.ENSDARP00000022305 |
| chmp5b | 7955.ENSDARP00000061395 |
| cldn10a | 7955.ENSDARP00000076338 |
| coa3 | 7955.ENSDARP00000098911 |
| cox7a2a | 7955.ENSDARP00000069708 |
| cry2a | 7955.ENSDARP00000090995 |
| ctdp1 | 7955.ENSDARP00000097776 |
| ctu2 | 7955.ENSDARP00000068045 |
| cux1a | 7955.ENSDARP00000075613 |
| cux1b | 7955.ENSDARP00000063328 |
| cxcr4a | 7955.ENSDARP00000074800 |
| dctd | 7955.ENSDARP00000053762 |
| dctn3 | 7955.ENSDARP00000060634 |
| ddx51 | 7955.ENSDARP00000029445 |
| ddx54 | 7955.ENSDARP00000108944 |
| dock10 | 7955.ENSDARP00000128864 |
| dot1l | 7955.ENSDARP00000083509 |
| dscaml1 | 7955.ENSDARP00000074099 |
| dync2li1 | 7955.ENSDARP00000058146 |
| dzank1 | 7955.ENSDARP00000072635 |
| dzip1 | 7955.ENSDARP00000105423 |
| ebna1bp2 | 7955.ENSDARP00000071726 |
| eif2a | 7955.ENSDARP00000068657 |
| eif2s2 | 7955.ENSDARP00000069494 |
| elp3 | 7955.ENSDARP00000113240 |
| etv5b | 7955.ENSDARP00000107613 |
| evpla | 7955.ENSDARP00000021688 |
| exd1 | 7955.ENSDARP00000112189 |
| fam213b | 7955.ENSDARP00000113812 |
| fam69a | 7955.ENSDARP00000086003 |
| fancg | 7955.ENSDARP00000038408 |
| fbxo15 | 7955.ENSDARP00000113137 |
| fga | 7955.ENSDARP00000029074 |
| fgb | 7955.ENSDARP00000016228 |
| fra10ac1 | 7955.ENSDARP00000112810 |
| fuca2 | 7955.ENSDARP00000064699 |
| gemin4 | 7955.ENSDARP00000060468 |
| gramd1a | 7955.ENSDARP00000100378 |
| grk7b | 7955.ENSDARP00000072364 |
| haus5 | 7955.ENSDARP00000056628 |
| hemk1 | 7955.ENSDARP00000102499 |
| hmha1 | 7955.ENSDARP00000083604 |
| hnrnpa1a | 7955.ENSDARP00000076883 |
| ighmbp2 | 7955.ENSDARP00000060290 |
| imp3 | 7955.ENSDARP00000091482 |
| isl1l | 7955.ENSDARP00000025628 |
| katnbl1 | 7955.ENSDARP00000062349 |
| kiaa0947l | 7955.ENSDARP00000122110 |
| kif6 | 7955.ENSDARP00000094438 |
| lace1b | 7955.ENSDARP00000105122 |
| lrp2a | 7955.ENSDARP00000108215 |
| lsm4 | 7955.ENSDARP00000037344 |
| map3k4 | 7955.ENSDARP00000069816 |
| mbnl1 | 7955.ENSDARP00000088834 |
| mccc1 | 7955.ENSDARP00000070658 |
| mdn1 | 7955.ENSDARP00000025022 |
| megf11 | 7955.ENSDARP00000085288 |
| mep1a.2 | 7955.ENSDARP00000009962 |
| mier3 | 7955.ENSDARP00000092379 |
| minpp1b | 7955.ENSDARP00000016794 |
| miox | 7955.ENSDARP00000115890 |
| mtf2 | 7955.ENSDARP00000109280 |
| mthfd1b | 7955.ENSDARP00000020770 |
| mylk3 | 7955.ENSDARP00000102547 |
| n6amt1 | 7955.ENSDARP00000051918 |
| ncf2 | 7955.ENSDARP00000024621 |
| ndufb4 | 7955.ENSDARP00000043608 |
| nek8 | 7955.ENSDARP00000067094 |
| nfu1 | 7955.ENSDARP00000114381 |
| nhsl1b | 7955.ENSDARP00000062531 |
| nkiras1 | 7955.ENSDARP00000043219 |
| nom1 | 7955.ENSDARP00000122404 |
| nthl1 | 7955.ENSDARP00000062940 |
| nup133 | 7955.ENSDARP00000023984 |
| nup37 | 7955.ENSDARP00000018434 |
| nup88 | 7955.ENSDARP00000025718 |
| nvl | 7955.ENSDARP00000032193 |
| nyap2b | 7955.ENSDARP00000122586 |
| odf3l2 | 7955.ENSDARP00000027071 |
| ogfod2 | 7955.ENSDARP00000073125 |
| oit3 | 7955.ENSDARP00000107933 |
| olfcw1 | 7955.ENSDARP00000120816 |
| or135-1 | 7955.ENSDARP00000070197 |
| p2rx8 | 7955.ENSDARP00000038849 |
| parp3 | 7955.ENSDARP00000026625 |
| pdcd2 | 7955.ENSDARP00000017163 |
| pdpk1a | 7955.ENSDARP00000093021 |
| pdpk1b | 7955.ENSDARP00000019085 |
| pes | 7955.ENSDARP00000026992 |
| pet112 | 7955.ENSDARP00000093298 |
| pex11g | 7955.ENSDARP00000066167 |
| pex6 | 7955.ENSDARP00000095384 |
| phax | 7955.ENSDARP00000065035 |
| phf14 | 7955.ENSDARP00000082096 |
| popdc2 | 7955.ENSDARP00000092923 |
| ppap2b | 7955.ENSDARP00000078333 |
| ppil4 | 7955.ENSDARP00000064389 |
| ppp2r3b | 7955.ENSDARP00000104429 |
| prkd3 | 7955.ENSDARP00000101844 |
| pum2 | 7955.ENSDARP00000081013 |
| pus10 | 7955.ENSDARP00000097545 |
| rbl2 | 7955.ENSDARP00000067923 |
| rpl11 | 7955.ENSDARP00000063869 |
| rplp0 | 7955.ENSDARP00000067953 |
| rtn4ip1 | 7955.ENSDARP00000040031 |
| rufy2 | 7955.ENSDARP00000083566 |
| sap30bp | 7955.ENSDARP00000039877 |
| sb:cb472 | 7955.ENSDARP00000069215 |
| sbf2 | 7955.ENSDARP00000077099 |
| scg3 | 7955.ENSDARP00000105698 |
| sdr39u1 | 7955.ENSDARP00000036413 |
| sesn2 | 7955.ENSDARP00000093160 |
| setd8b | 7955.ENSDARP00000109584 |
| setmar | 7955.ENSDARP00000101881 |
| sgsm1b | 7955.ENSDARP00000060945 |
| si:ch1073-145m9.1 | 7955.ENSDARP00000128942 |
| si:ch211-131k2.3 | 7955.ENSDARP00000128587 |
| si:ch211-237c6.4 | 7955.ENSDARP00000076301 |
| si:ch211-262h13.5 | 7955.ENSDARP00000095210 |
| si:dkey-256h2.1 | 7955.ENSDARP00000033960 |
| siae | 7955.ENSDARP00000059310 |
| slc12a7b | 7955.ENSDARP00000083665 |
| slc26a6l | 7955.ENSDARP00000094609 |
| slc34a2a | 7955.ENSDARP00000023797 |
| slc52a2 | 7955.ENSDARP00000074710 |
| slc5a2 | 7955.ENSDARP00000040379 |
| sqstm1 | 7955.ENSDARP00000117113 |
| ss18l2 | 7955.ENSDARP00000058159 |
| ssr1 | 7955.ENSDARP00000108188 |
| sts | 7955.ENSDARP00000069736 |
| styk1a | 7955.ENSDARP00000095367 |
| swsap1 | 7955.ENSDARP00000120384 |
| sypb | 7955.ENSDARP00000082673 |
| tbc1d31 | 7955.ENSDARP00000099918 |
| tbcd | 7955.ENSDARP00000105984 |
| tbx24 | 7955.ENSDARP00000015108 |
| tceanc2 | 7955.ENSDARP00000085930 |
| tmem123 | 7955.ENSDARP00000122717 |
| tmem14cb | 7955.ENSDARP00000065335 |
| tmod1 | 7955.ENSDARP00000103325 |
| tnnt1 | 7955.ENSDARP00000044153 |
| top2a | 7955.ENSDARP00000096930 |
| tpc3 | 7955.ENSDARP00000098968 |
| trim36 | 7955.ENSDARP00000085610 |
| trim37 | 7955.ENSDARP00000117221 |
| trim46 | 7955.ENSDARP00000045890 |
| trmt2b | 7955.ENSDARP00000112957 |
| ttc21b | 7955.ENSDARP00000044269 |
| ttc36 | 7955.ENSDARP00000017354 |
| txnrd2.2 | 7955.ENSDARP00000103469 |
| ulk2 | 7955.ENSDARP00000128554 |
| umodl1 | 7955.ENSDARP00000090626 |
| unm_sa821 | 7955.ENSDARP00000101177 |
| uox | 7955.ENSDARP00000026870 |
| upp2 | 7955.ENSDARP00000053472 |
| uqcc3 | 7955.ENSDARP00000121818 |
| v2rx1 | 7955.ENSDARP00000026867 |
| wdpcp | 7955.ENSDARP00000129643 |
| wdr78 | 7955.ENSDARP00000065205 |
| wdr96 | 7955.ENSDARP00000123525 |
| wfikkn1 | 7955.ENSDARP00000098282 |
| wu:fj04c06 | 7955.ENSDARP00000073798 |
| xrcc1 | 7955.ENSDARP00000023168 |
| xrcc3 | 7955.ENSDARP00000004619 |
| xylb | 7955.ENSDARP00000063503 |
| zgc:110366 | 7955.ENSDARP00000127098 |
| zgc:112285 | 7955.ENSDARP00000069834 |
| zgc:123217 | 7955.ENSDARP00000097428 |
| zgc:152986 | 7955.ENSDARP00000103646 |
| zgc:153650 | 7955.ENSDARP00000018562 |
| zgc:158645 | 7955.ENSDARP00000114794 |
| zgc:161973 | 7955.ENSDARP00000043955 |
| zgc:171489 | 7955.ENSDARP00000068618 |
| zgc:174164 | 7955.ENSDARP00000074097 |
| zgc:193811 | 7955.ENSDARP00000101346 |
| zgc:64190 | 7955.ENSDARP00000067754 |
| zmynd8 | 7955.ENSDARP00000040234 |
| znf511 | 7955.ENSDARP00000106376 |
| zp3b | 7955.ENSDARP00000058255 |
| zte38 | 7955.ENSDARP00000053866 |

Table S8. Table of protein summaries for all single copy orthologs with evidence for episodic selection in Spikedace and Loach Minnow. Listed are the identifiers and gene names for the ortholog present in the Zebrafish (*Danio rerio*). All identifiers and gene names are appended from Entrez based on the NCBI protein ID.

| **GO-term** | **Description** | **Count in Network** | **Strength** | **P-value** |
| --- | --- | --- | --- | --- |
| GO:0042254 | Ribosome biogenesis | 13 of 284 | 0.67 | 0.0086 |
| GO:0034660 | ncRNA metabolic process | 18 of 417 | 0.65 | 0.0026 |
| GO:0034470 | ncRNA processing | 14 of 339 | 0.63 | 0.0104 |
| GO:0090304 | Nucleic acid metabolic process | 41 of 1940 | 0.34 | 0.0066 |
| GO:0006139 | Nucleobase-containing compound metabolic process | 49 of 2474 | 0.31 | 0.0048 |
| GO:0046483 | Heterocycle metabolic process | 50 of 2614 | 0.3 | 0.0066 |
| GO:0006725 | Cellular aromatic compound metabolic process | 50 of 2662 | 0.29 | 0.007 |
| GO:0034641 | Cellular nitrogen compound metabolic process | 57 of 3180 | 0.27 | 0.0066 |
| GO:1901360 | Organic cyclic compound metabolic process | 51 of 2875 | 0.26 | 0.0183 |
| GO:0071840 | Cellular component organization or biogenesis | 85 of 5882 | 0.17 | 0.0396 |
| GO:0008152 | Metabolic process | 132 of 9636 | 0.15 | 0.0035 |

Table S9. List of enriched terms using all Zebrafish ontology databases in STRING-db. Listed are all significantly enriched terms sorted by strength of the interaction among genes. All p-values are corrected for the false discovery rate (FDR). Strength measures the magnitude of the enrichment using the log (base 10) ratio between the number of proteins in our network with an annotation term and the number of proteins expected with the same annotation term in a network of the same size.

**
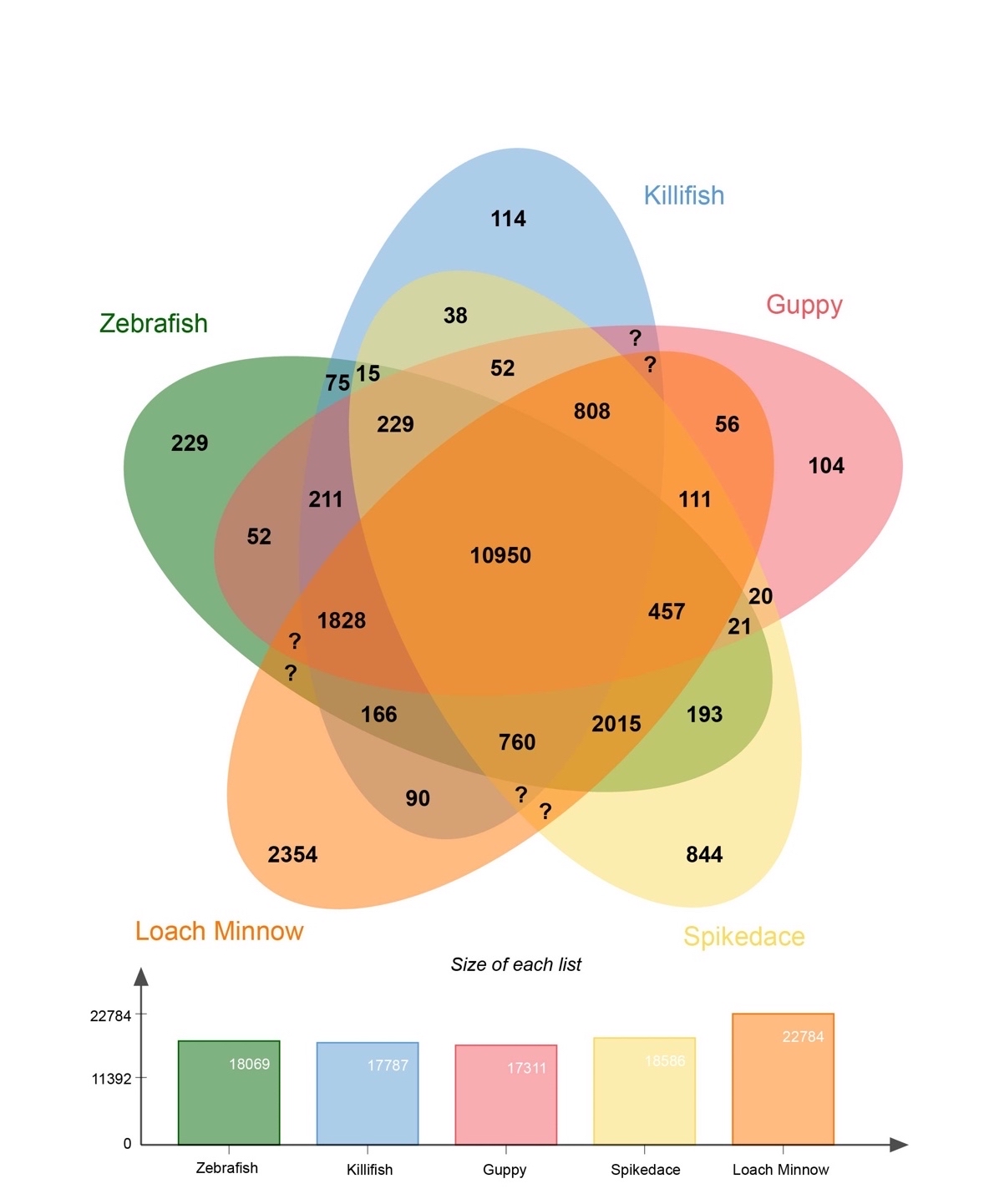
**

Figure S1. Output of OrthoVenn2 displaying the number of shared and unique orthologs between Spikedace, Loach Minnow, and three other model fish species. A. Venn-diagram showing the overlap in the number of identified orthologs between protein annotations for each assembly. B. Counts in the number of identified orthologous clusters found using protein annotations from each assembly.

Counts of Orthologous Elements

B.

A.


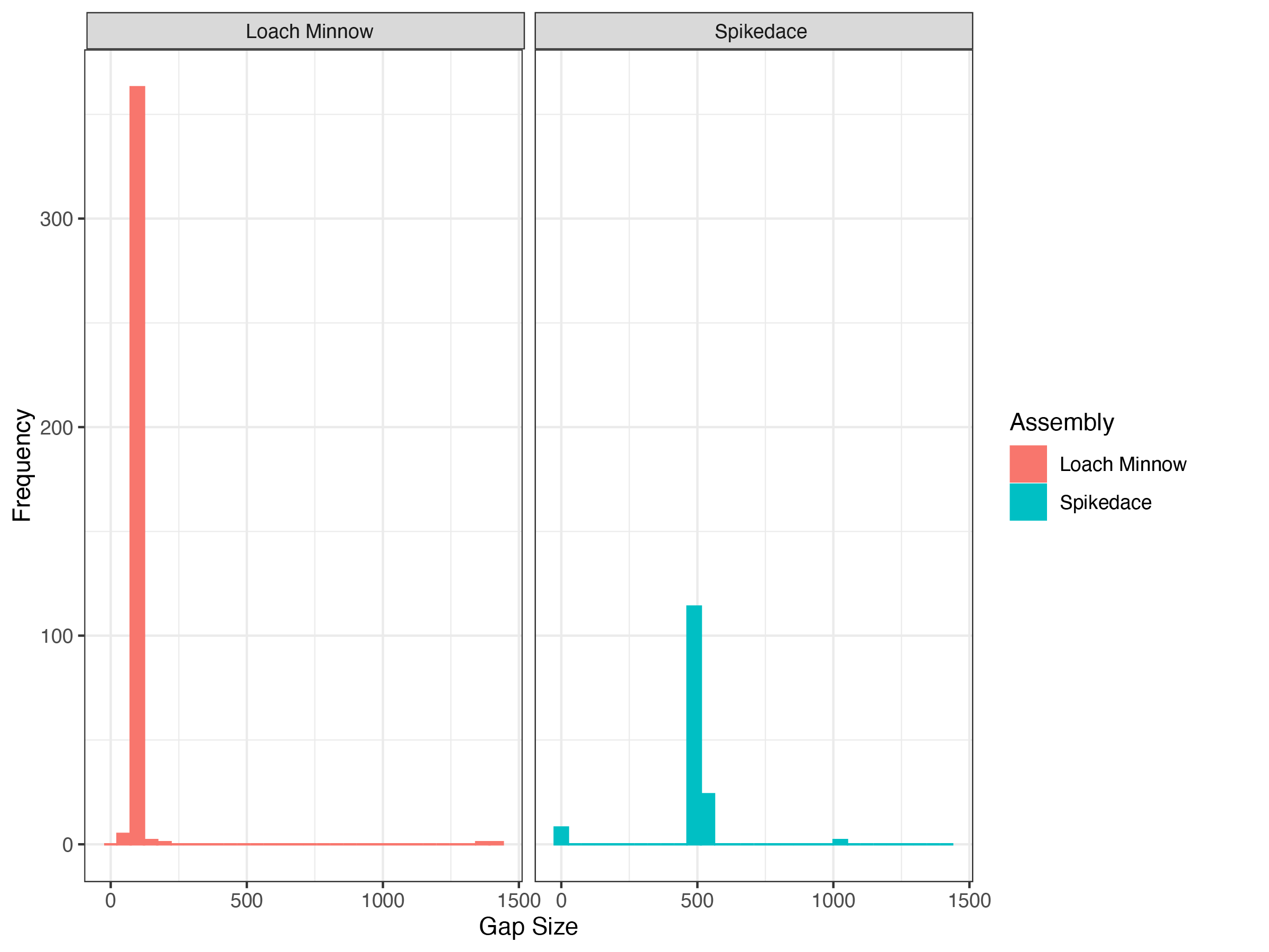


Figure S2. Gap size distribution for each genome assembly.


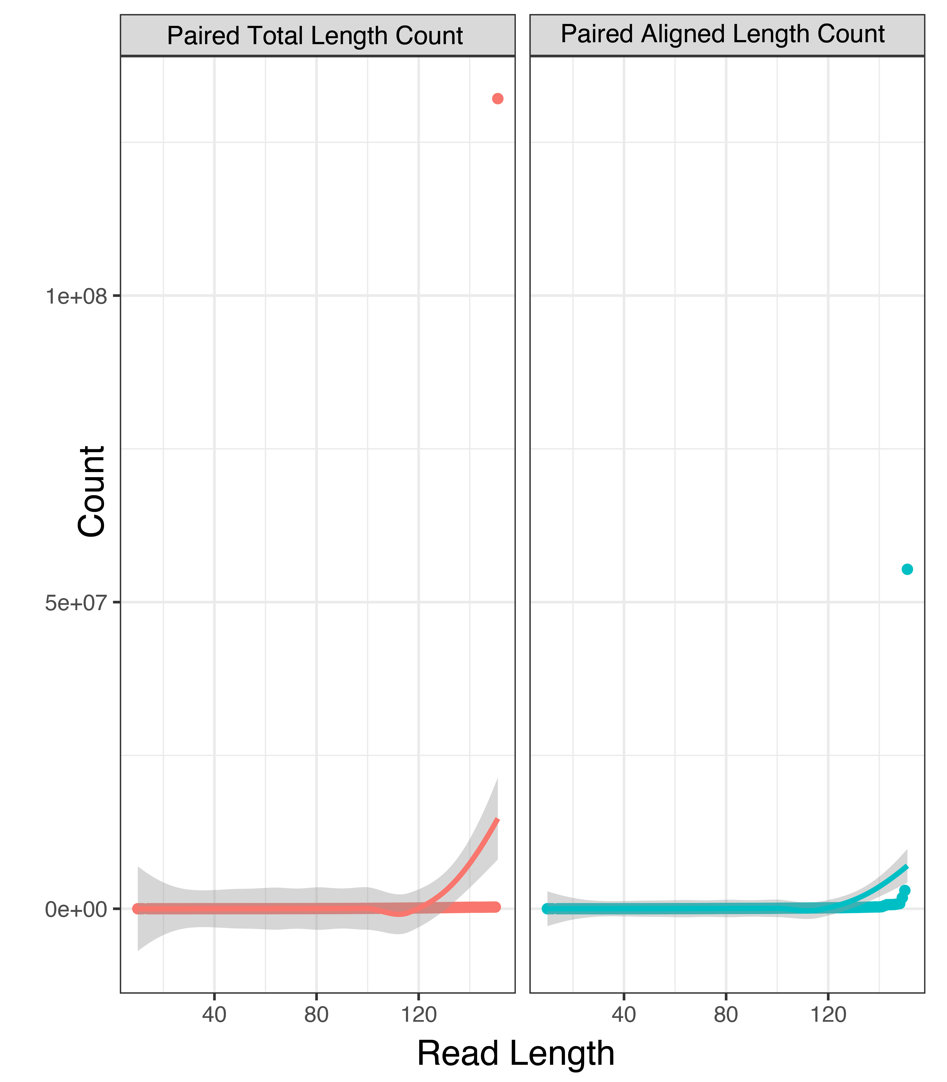


Figure S3. Counts of paired-end RNASeq reads aligned using STAR. All reads of length zero were removed prior to plotting.

Figure S4. Percent coverage of each genome as a function repeat class/family. The length of each sequence belonging to a class or family of repeat was summed and divided by the total genome size. Excluding unknown repeats, the Loach Minnow genome primarily contained simple repeats, while both Spikedace and Loach Minnow contained a large proportion of LTR/DIRS repeats.

Figure S5. The distributions of (A) transcript lengths, (B) exons per transcript, (C) introns per transcript, and (D) length of CDS sequences in Spikedace (blue) and Loach Minnow (green) genomes.


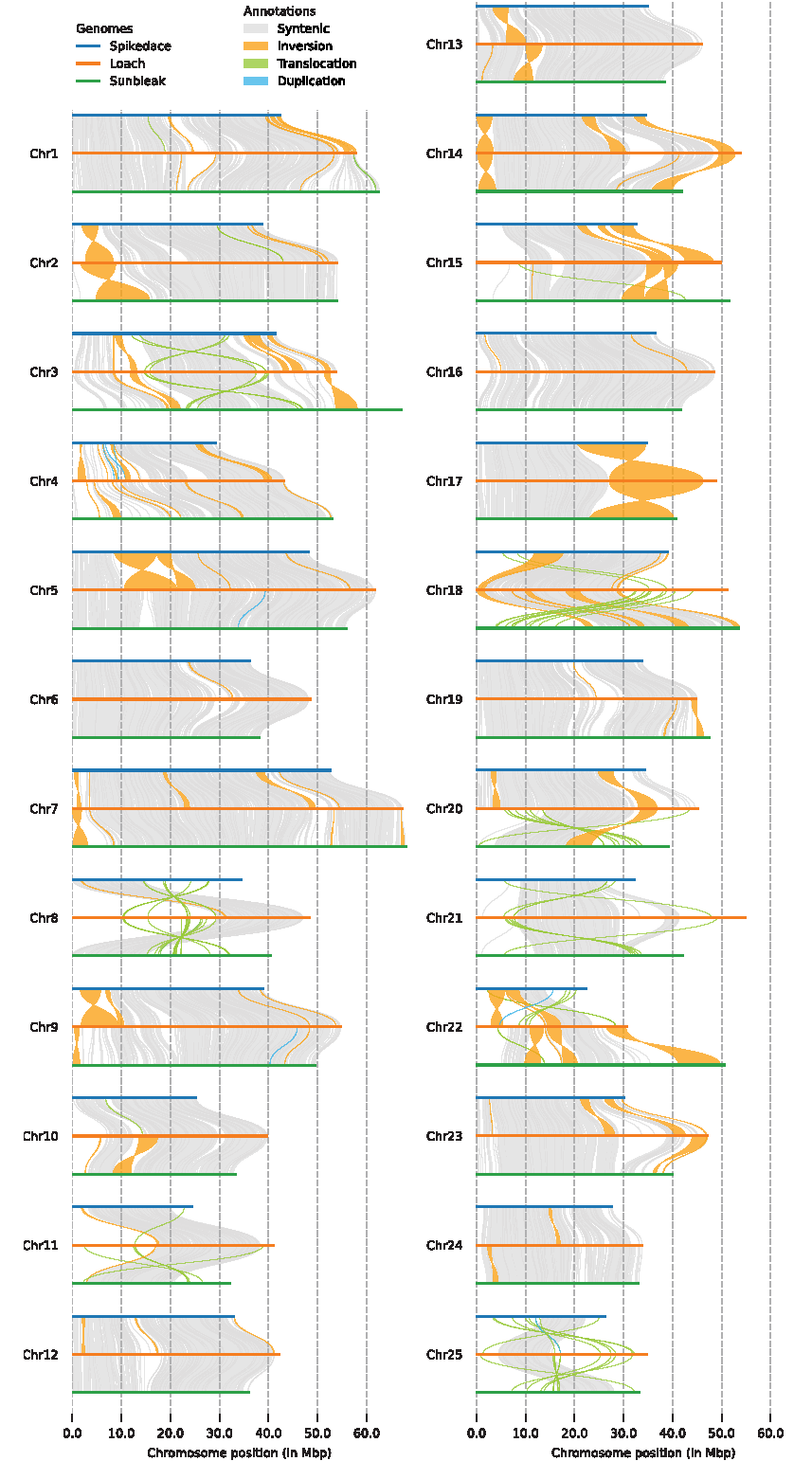


Figure S6. Synteny analysis of all 25 chromosomes (horizontal lines) in Spikedace (blue) Loach Minnow (orange) and Sunbleak (green) genome assemblies indicates high levels of similarity between these leuciscid taxa. Gray, light orange, light green, and light blue lines connecting the assemblies indicate regions annotated as syntenic, inversions, translocations, or duplications, respectively.

**Table and Figure Captions**

Table S1. All installed software packages used for both genomes along with the relevant versions.

Table S2. Candidate contigs containing mitochondrial or bacterial contamination along with alignment length and percent of reference scaffold covered.

Table S3. Output of RepeatMasker showing the total number of repeats by repeat class for Loach Minnow.

Table S4. Output of RepeatMasker showing the total number of repeats by repeat class for Spikedace.

Table S5. Summary statistics for the distribution of gap sizes in each genome assembly.

Table S6. Number and total length of syntenic, structural rearrangement, and local variation regions. SYN: syntenic region; INV: inversion; TRN: translocation; DUP-REF: duplication found in the reference but not query genome; DUP-QRY: duplication found in the query but not reference genome; UNQ-REF: unaligned regions specific to the reference; UNQ-QRY: unaligned regions specific to the query; SNPs: single nucleotide polymorphisms; INS: insertions; DEL: deletions; CPG: copy gain variation; CPL: copy loss variation.

Table S7. Results of gene family expansion analysis for the largest gene families with at least a single ortholog in zebrafish. We provide the number of genes in each orthogroup for Spikedace and Loach Minnow (de novo counts), number of Zebrafish orthologues, corresponding zebrafish gene symbols and sequence IDs within gene families. We also provided enriched GO terms and protein domains. For gene family expansions in both species we provide the sum of Spikedace and Loach Minnow and individual counts in parentheses (Spikedace:LoachMinnow).

Table S8. Table of protein summaries for all single copy orthologs with evidence for episodic selection in Spikedace and Loach Minnow. Listed are the identifiers and gene names for the ortholog present in the Zebrafish (*Danio rerio*). All identifiers and gene names are appended from Entrez based on the NCBI protein ID.

Table S9. List of enriched terms using all Zebrafish ontology databases in STRING-db. Listed are all significantly enriched terms sorted by strength of the interaction among genes. All p-values are corrected for the false discovery rate (FDR). Strength measures the magnitude of the enrichment using the log (base 10) ratio between the number of proteins in our network with an annotation term and the number of proteins expected with the same annotation term in a network of the same size.

Figure S1. Output of OrthoVenn2 displaying the number of shared and unique orthologs between Spikedace, Loach Minnow, and three other model fish species. A. Venn-diagram showing the overlap in the number of identified orthologs between protein annotations for each assembly. B. Counts in the number of identified orthologous clusters found using protein annotations from each assembly.

Figure S2. Gap size distribution for each genome assembly.

Figure S3. Counts of paired-end RNASeq reads aligned using STAR. All reads of length zero were removed prior to plotting.

Figure S4. Percent coverage of each genome as a function repeat class/family. The length of each sequence belonging to a class or family of repeat was summed and divided by the total genome size. Excluding unknown repeats, the Loach Minnow genome primarily contained simple repeats, while both Spikedace and Loach Minnow contained a large proportion of LTR/DIRS repeats.

Figure S5. The distributions of (A) transcript lengths, (B) exons per transcript, (C) introns per transcript, and (D) length of CDS sequences in Spikedace (blue) and Loach Minnow (green) genomes.

Figure S6. Synteny analysis of all 25 chromosomes (horizontal lines) in Spikedace (blue) Loach Minnow (orange) and Sunbleak (green) genome assemblies indicates high levels of similarity between these *Cyprinidae* taxa. Gray, light orange, light green, and light blue lines connecting the assemblies indicate regions annotated as syntenic, inversions, translocations, or duplications, respectively.
